# Gene expression of putative type VI secretion system (T6SS) genes in the emergent fish pathogen *Francisella noatunensis* subsp. *orientalis* in different physiochemical conditions

**DOI:** 10.1186/s12866-019-1389-7

**Published:** 2019-01-21

**Authors:** Jainee Lewis, Esteban Soto

**Affiliations:** Department of Medicine and Epidemiology, University of California-Davis, School of Veterinary Medicine, Davis, CA 95616 USA

**Keywords:** Francisella, Gene expression, Oxidative stress, Type six secretion system

## Abstract

**Background:**

*Francisella noatunensis* subsp. *orientalis* (*Fno*) is an emergent fish pathogen and the etiologic agent of piscine francisellosis. Besides persisting in the environment in both biofilm and planktonic forms, *Fno* is known to infect and replicate inside tilapia macrophages and endothelial-derived cells. However, the mechanism used by this emergent bacterium for intracellular survival is unknown. Additionally, the basis of virulence for *Fno* is still poorly understood. Several potential virulence determinants have been identified in *Fno,* including homologues of the recently described *F. tularensis* Type VI Secretion System (T6SS). In order to gain a better understanding of the role the putative *Fno* T6SS might play in the pathogenesis of piscine francisellosis, we performed transcriptional analysis of *Fno* T6SS gene-homologues under temperature, acidic, and oxidative stress conditions.

**Results:**

Few transcriptional differences were observed at different temperatures, growth stages and pHs; however, a trend towards higher expression of *Fno* T6SS-homologue genes at 25 °C and under oxidative stress was detected when compared to those quantified at 30 °C and under no H_2_O_2_ (*p* < 0.05).

**Conclusions:**

Results from this study suggest that several of the *F. tularensis* T6SS-homologues may play an important role in the virulence of *Fno,* particularly when the bacterium is exposed to low temperatures and oxidative stress.

**Electronic supplementary material:**

The online version of this article (10.1186/s12866-019-1389-7) contains supplementary material, which is available to authorized users.

## Background

Members of the genus *Francisella* are small, Gram-negative, pleomorphic, non-motile coccobacilli in the gamma-Proteobacteria family Francisellaceae, order Thiotrichales. The known diversity within the *Francisella* genus has expanded significantly from two major species groups, *F. tularensis* [1] and *F. philomiragia* [2], to over 6 species in the genus, some causing important diseases in aquatic animals [3–7].

*Francisella noatunensis,* an emerging pathogen of fish, is the causative agent of piscine francisellosis. Of the fish pathogenic *Francisella* sp. there are two distinct genetic lineages: *Francisella noatunensis* subsp. *noatunensis* (*Fnn*) and *Francisella noatunensis* subsp. *orientalis* (*Fno*). *Fnn* infects cold-water fish species and was first identified in diseased cultured cod (*Gadus morhua* L.) from Norway [8]. *Fno* causes diseases in warm-water fish species and was first identified in three-line grunt (*Parapristipoma trilineatum*) from Japan [9]. In the last decade, piscine francisellosis has been diagnosed worldwide in cultured and wild fish in marine and freshwater environments reaching mortalities of 30–75% [3, 6–10].

Piscine francisellosis due to *Fno* is characterized by a severe granulomatous inflammatory response in multiple organs, particularly in the kidney and spleen [6, 7]. Little information has been published regarding the epizootiology of piscine francisellosis. Environmental factors such as temperature play an important role in the development of piscine francisellosis [6, 7, 11]. Higher mortalities typically occur in cooler water temperatures < 28 °C with few to no mortalities occurring at temperatures > 28 °C degrees [6, 7, 11].

Unlike *F. tularensis*, the etiologic agent of tularemia in humans and mammals, *Fno* does not grow above 30 °C and therefore is not associated with disease in warm-blooded animals. However, the genomes of *F. tularensis* and *F. noatunensis* are very similar, particularly for the region encoding the *Francisella* pathogenicity island [12]. Importantly, *F. noatunensis* colonizes and replicates in phagocytes and endothelial cells in a process that is analogous to that of *F. tularensis* in mammalian phagocytes [13–16].

Many have explored the internalization and intracellular trafficking and survival used by *F. tularensis* in mammalian cells. Our current understanding indicates that *Francisella tularensis* enters host macrophages in asymmetric spacious pseudopod loops [17], arrests maturation of the phagosome at a late endosomal-like stage, and in minutes, escapes into the cytosol of the host cells were it replicates [18, 19] eventually inducing apoptosis [20] and pyroptosis [21]. Although *Fno* and *Fnn* have been found to survive inside fish macrophages and endothelial cells [15, 16, 22], the basis of virulence for *Fno* and the pathogenesis of piscine francisellosis are still poorly understood. Additionally, histological and electron microscopic analysis suggest that a potentially different mechanism for intracellular survival is utilized by *Fno* as compared with the previously described mechanism used by *F. tularensis*, since most intracellular *Fno* are typically observed in spacious vacuoles within the macrophages, not in the cytoplasm as *F. tularensis* [15, 16, 23].

Several virulence determinants have been identified in *Fno,* including a homologous pathogenicity island (PI) to the *F. tularensis* PI [24, 25]. Additionally, genes potentially encoding proteins similar to components of the *F. tularensis* type VI secretion system (T6SS) have been identified in *Fno* including IglA, IglB, VgrG, DotU, and PdpB [25].

The T6SS is a recently described bacterial secretion system that translocates proteins into a variety of recipient cells including eukaryotic cell targets and other bacteria [25]. T6SSs are very large with up to thirteen proteins that appear to be well conserved and are thought to play a structural role in the secretion apparatus. Structural components of the T6SS apparatus may also serve as effector proteins [25]. These effector proteins are thought to have many functions with many directed against the cell wall and membrane of other neighboring bacteria that may be competing to exploit a specific host niche [25]. T6SSs are also thought to play a role in bacteria-bacteria interactions that may ultimately increase the fitness of the T6SS expressing bacteria within host-associated microbial communities [25].

de Bruin et al., [26] demonstrate that *F. tularensis* deletion mutants of *iglABCD* and T6SS homologues *vgrG*, *dotU*, and *pdpB* were attenuated, and that those genes were all essential for intracellular growth and virulence. They proposed IglC as the analogue of Hcp, IglA and IglB as structural components of an outer tube that surrounds IglC subunits; and DotU and PdpB, IcmH and IcmF homologues, respectively, localizing to the inner membrane. Two of the proteins, VgrG and IglI, encoded in the *Francisella* PI have been shown to be required for *F. tularensis* phagosomal escape, intramacrophage growth and virulence in mice [26]. VgrG also doesn’t require the other FPI proteins, indicating that it acts in an FPI-independent manner. However VgrG, IglI and other FPI genes (including PdpB) are required for the secretion of IglI into the macrophage cytosol suggesting that VgrG and other FPI factors are components of a secretion system [26].

Few studies have investigated virulence factors in *Fno*. The *iglABCD* operon has been found important for virulence determination in *Fno* and necessary for induction of disease and intra-macrophage survival in tilapia and zebrafish [15, 16, 27, 28]. Hansen et al. [27] and Farrell et al. [28] demonstrated that mutation of the *pdpA* gene in *Fno* resulted in significant attenuation in the zebrafish and the hybrid red tilapia model of infection. Similarly, mutation of *clpB,*the gene encoding the Caseinolytic protease B in *Fnn,* caused in vitro and in vivo attenuation in the zebrafish [29].

Since temperature has been shown to play an important role in the development of piscine francisellosis, and since acidic and oxidative stress have been reported as important cues for virulence gene expression in multiple bacteria, we performed transcriptional analysis of the *Fno* T6SS-homologue genes with the goal of gaining a better understanding on this emergent disease pathogenesis.

## Results

### *Francisella noatunensis* subsp. *orientalis* growth at different temperatures

Similar growth patterns were observed at 25 °C and 30 °C during the first 30 h (Additional file 1 Figure S1). Post hoc comparisons showed that the two temperatures approached significance at time 30 h (*p* = 0.054), and then were significantly different at time 31 h (*p* = 0.0249), time 32 h (*p* = 0.0099), time 33 h (*p* = 0.0018), and time 34 h (*p* = 0.0006). Beginning at time 35 h, and for all subsequent times through time 90 h, the two temperatures were significantly different with *p* < 0.0001.

### Expression of *Fno* T6SS-homologue genes at different growth stages at different temperatures

The gene expression of putative T6SS genes was assessed during the exponential and stationary growth phases of *Fno* at 25 °C and 30 °C. A trend towards higher expression at 25 °C compared to 30 °C was observed in several experiments as all tested genes presented lower expression during stationary phase at the higher temperature (Table 1). Additionally, most presented lower expression during exponential phase at 30 °C when compared to exponential phase at 25 °C. Interestingly, *pdpB* showed significantly higher expression during exponential phase at 30 °C when compared to exponential phase at 25 °C (*p* ≤ 0.05) (Table 1). There were similar expression patterns at exponential and stationary phases when incubated at 25 °C; but a trend towards lower expression in stationary phase was observed when incubated at 30 °C (Table 1).

**Table 1:**
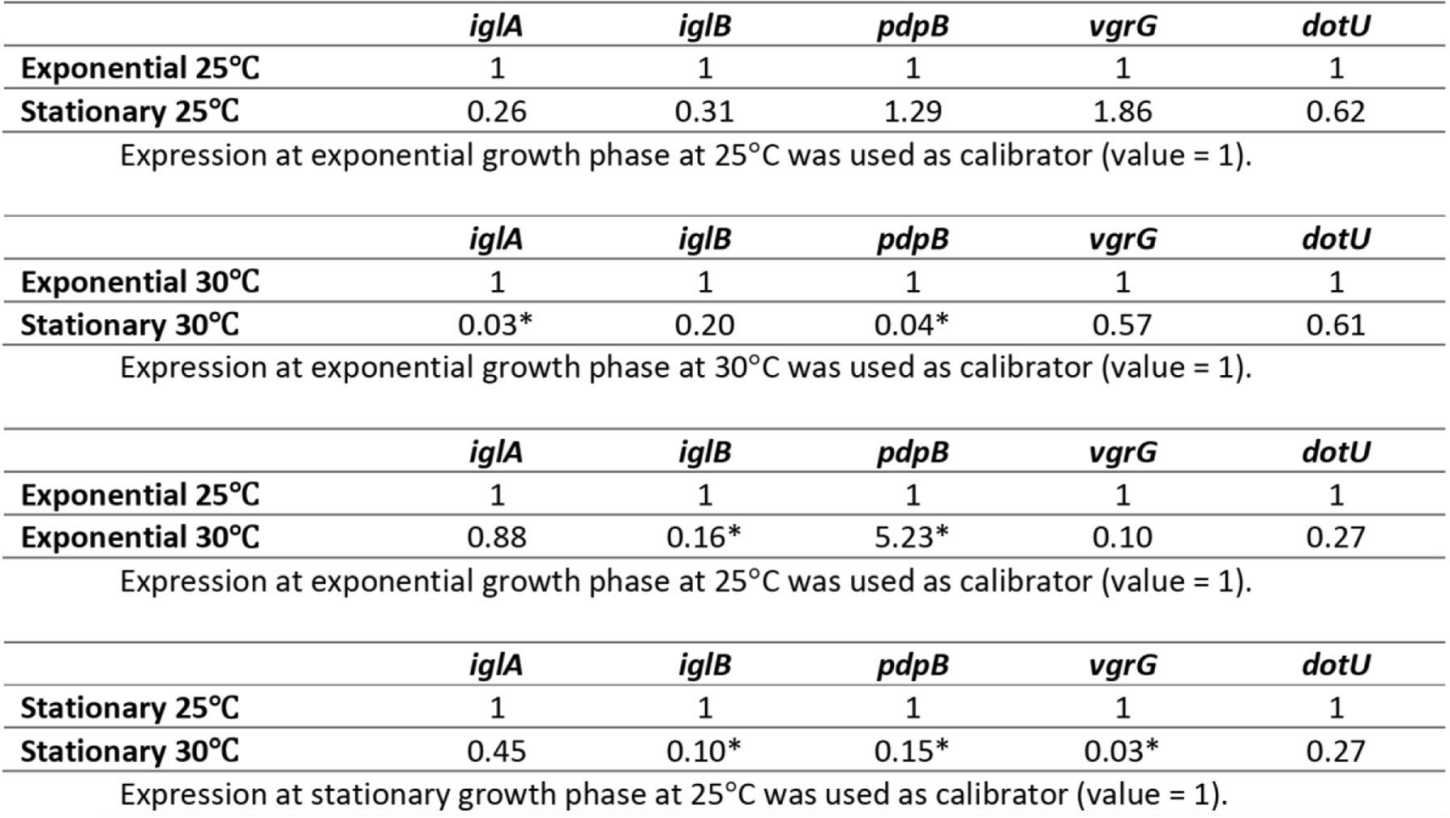
Expression profile of *Francisella noatunensis* subsp. *orientalis* putative T6SS genes, *iglA, iglB, pdpB, vgrG,* and *dotU*, during exponential and stationary growth phases when incubated at 25 or 30 °C. Gene expression was determined by qRT- PCR, using relative quantification. Gene expression was normalized by the housekeeping gene *ftsZ*

In some experiments exponential growth phase at 25 °C, exponential growth phase at 30 °C, or stationary growth phase at 25 °C were used as calibrator (value =1). Statistically significant differences relative to calibrator are marked (*, *P* < 0.05)

### Survival of *Fno* and expression of *Fno* T6SS-homologue genes at acidic pHs

**Fig. 1:**
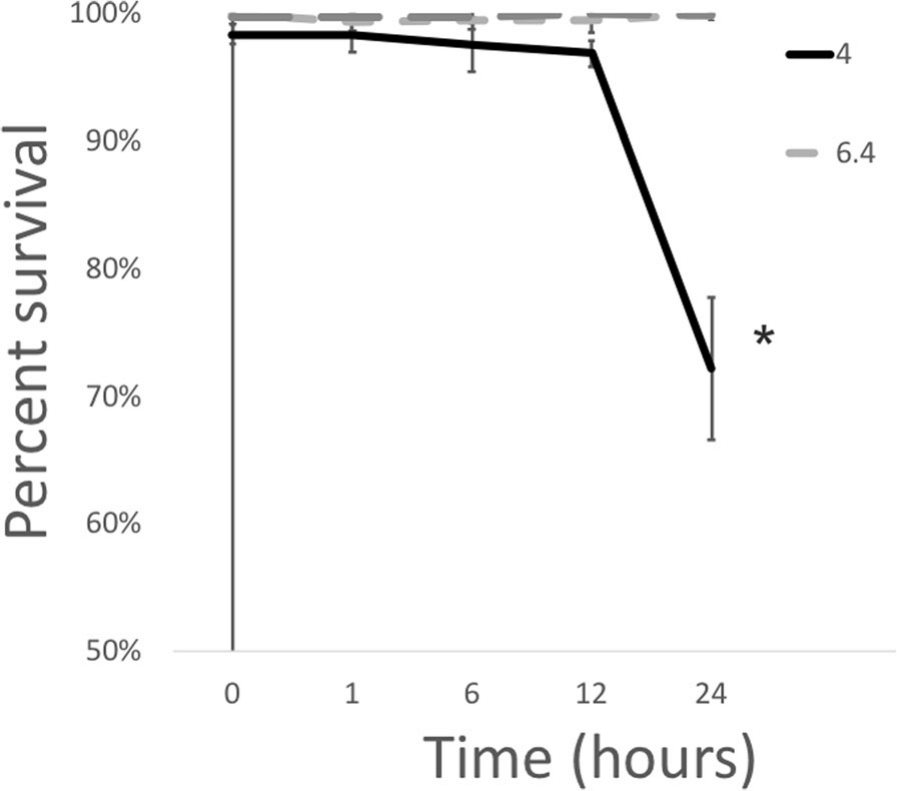
Percent survival of *Francisella noatunensis subsp. orientalis* at various pH in broth. Percent survival was calculated by comparing the number of bacteria in the test wells to the number of bacteria incubated for the same period of time at pH 7 (100% survival). The error bars represent the standard deviation of twelve replicate wells from three independent experiments

**Fig. 2:**
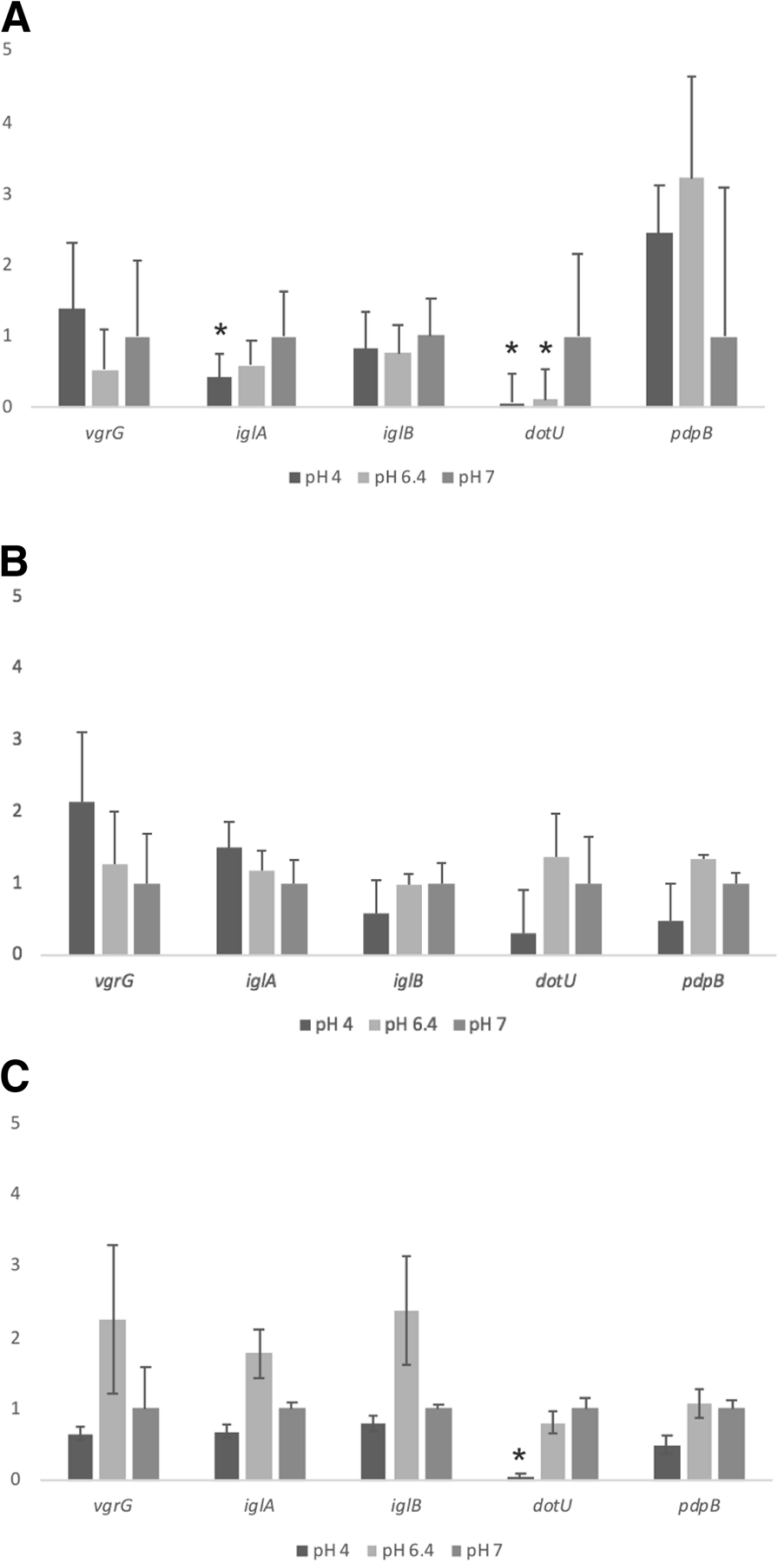
Expression profile of *Francisella noatunensis* subsp. *orientalis* putative T6SS genes, *iglA, iglB, pdpB, vgrG,* and *dotU* when exposed to different pHs for 1 h (**a**), 6 h (**b**) or 24 h (**c**). Gene expression was determined by qRT-PCR, using relative quantification. Gene expression was normalized by the housekeeping gene *ftsZ*. Zero hour of growth at pH 7 was used as calibrator (value =1). The error bars represent the standard deviation of nine replicate samples from three independent experiments. Statistically significant differences relative to 0 h at pH 7 are marked (*, *P* < 0.05)

In order to determine whether acidic stress could be the signal that leads to an expression of the *Fno* T6SS-homologue genes during infection, we first evaluated the survival of *Fno* in broth adjusted to pH 4.0, 6.4, and 7, and compared the expression of T6SS-homologues at each condition during 24 h. *Francisella noatunensis* subsp. *orientalis* was recovered at all conditions tested, however significantly fewer culturable organisms were quantified at 24 h at pH 4 when compared to those at neutral and slightly acidic pH of 6.4 (*p* ≤ 0.0001) (Fig. 1). Similar expression of the putative T6SS genes was observed at the different conditions and time points (Fig. 2); interestingly, significantly lower expression of *iglA* and *dotU* were detected under acidic conditions at some time points (Fig. 2).

### Survival of *Fno* and expression of *Fno* T6SS-homologue genes at different concentrations of H_2_O_2_

**Fig. 3:**
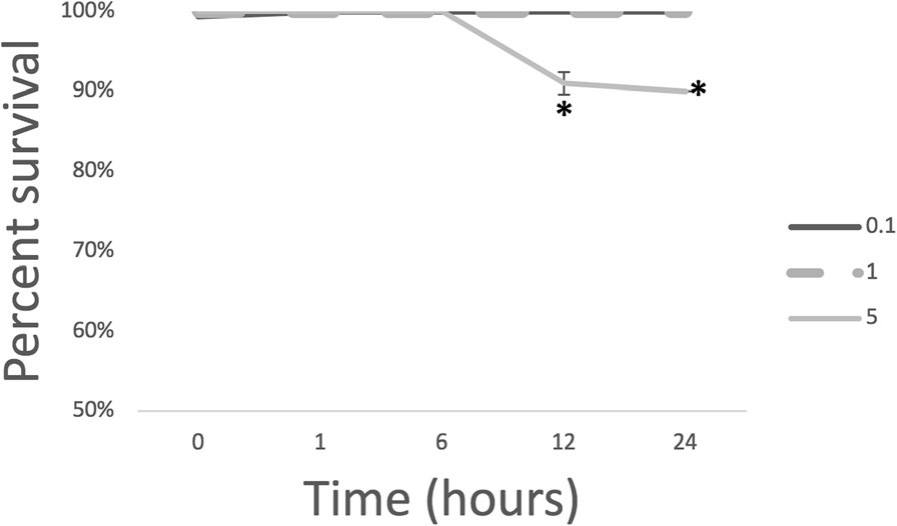
Percent survival of *Francisella noatunensis subsp. orientalis* at various hydrogen peroxide concentrations in broth. Percent survival was calculated by comparing the number of bacteria in the test wells to the number of bacteria incubated for the same period of time at 0 mM concentration of hydrogen peroxide. The error bars represent the standard deviation of twelve replicate wells from three independent experiments. Statistically significant differences relative to the same period of time at 0 mM are marked (*, *P* < 0.05)

In order to determine whether oxidative stress could be the signal that leads to an expression of the *Fno* T6SS-homologue genes during infection, we first evaluated the persistence of *Fno* in broth adjusted to 0.1 mM, 1 mM, and 5 mM H_2_O_2_, and compared the expression of T6SS-homologue at each condition during 24 h. Culturability of *Fno* decreased (*p* < 0.0001) over time when incubated at 5 mM H_2_O_2_. Similar persistence was found at all other concentrations (Fig. 3).

**Fig. 4:**
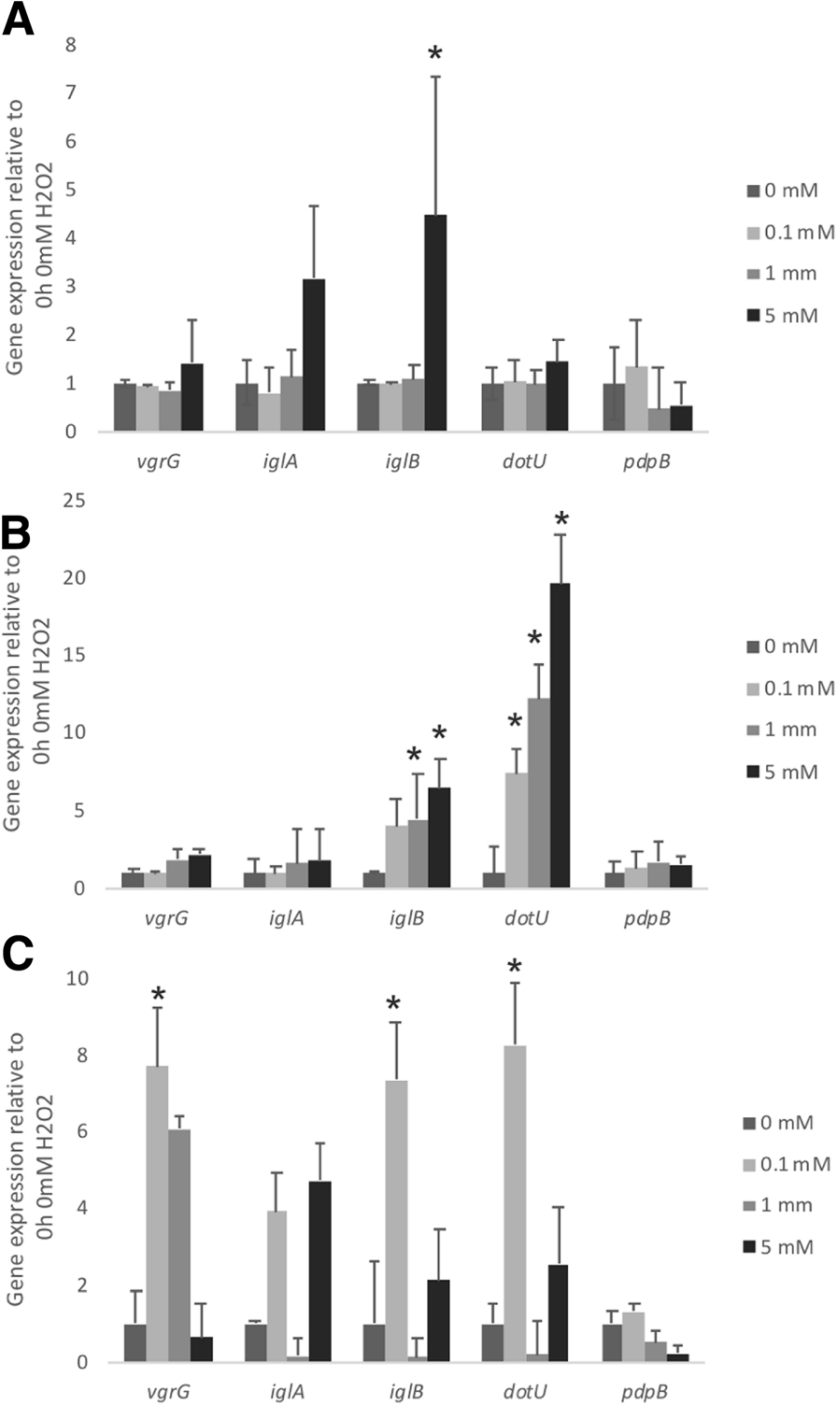
Expression profile of *Francisella noatunensis* subsp. *orientalis* putative T6SS genes, *iglA, iglB, pdpB, vgrG,* and *dotU* when exposed to different concentrations of hydrogen peroxide for 1 h (**a**), 6 h (**b**) or 24 h (**c**). Gene expression was determined by qRT-PCR, using relative quantification. Gene expression was normalized by the housekeeping gene *ftsZ*. Zero hour of growth at 0 mM was used as calibrator (value =1). The error bars represent the standard deviation of nine replicate samples from three independent experiments. Statistically significant differences relative to the same period of time at 0 mM are marked (*, *P* < 0.05)

A trend towards higher expression of putative T6SS genes was detected during oxidative stress (Fig. 4). The increased expression of the different T6SS-homologue genes was detected at different time points (Fig. 4).

## Discussion

Virulence gene expression in most bacteria is a highly regulated event, affected by a variety of parameters including temperature, growth phase, pH, and oxidative stress. In order to survive in different environments *Fno* has to be able to sense and respond to signals from its surroundings to precisely regulate expression of necessary genes, including those involved in attachment, intracellular replication and stress responses.

In the model of the T6SS apparatus that has been proposed by [25, 26], IglA and IglB form the outer tube of the T6SS apparatus with IglC being the dominant component of the inner tube. Additionally, PdpB and DotU appear to reside in the inner membrane and the stability of PdpB is dependent on the presence of DotU [25, 26]. Finally, VgrG appears to be secreted by the T6SS apparatus and serves as one of the effector proteins [25, 26]. In this study we sought to examine the effect of environmental stimuli such as temperature, bacterial growth phase, pH, and oxidative stress on the expression of putative T6SS genes of the fish pathogen *Fno*.

Similar growth of *Fno* was detected during the first 30 h at both temperatures; however, *Fno* grew significantly greater at 25 °C after 31 h of incubation (Additional file 1 Figure S1). Significantly greater expression of some of the T6SS-homologue genes was also observed at the lower temperature. These temperatures were chosen since previous in-vivo infectious challenges have demonstrated that tilapia are particularly susceptible to piscine francisellosis when the environmental temperature is < 30 °C, whereas no mortality, clinical presentation of diseases and lesions are found when fish are maintained at higher temperatures [11, 16]. Differential expression of virulence genes at environmental (25 °C) and mammalian host body temperature (37 °C) has also been reported for *F. tularensis* [30, 31].

Facultative and obligate intracellular pathogens utilize different mechanisms for intracellular survival. In some bacteria, phagosome acidification and oxidative stress induces the expression of virulence and stress-associated genes allowing pathogenic bacteria to escape the phagosome or persist even in the presence of oxidative and acidic stress [32–35]. Newly formed phagosomes are immature organelles that are unable to degrade and kill microorganisms. In macrophage and dendritic cells, phagosomes mature overtime by fusing to endosomes and lysosomes, which deliver various hydrolases and proteases to the lumen of the phagosome [36, 37]. A main event in phagosome maturation occurs when the phagosomal lumen is acidified to pH 4 to 5 by membrane-embedded ATP complexes, the vacuolar H^+^- ATPases. This creates an environment that is able to degrade and kill most microbes [38].

Several intracellular pathogens utilize vacuolar acidification as a cue for virulence gene expression. For example, the salmonid pathogen *Piscirickettsia salmonis*, expresses Dot/Icm T4SS genes at pH 4.0 [39]. Similar conditions are required for *Edwardsiella ictaluri* expression of the T3SS [40]; however, at the time points tested in the current study, acidic pH failed to induced expression of the *Fno* T6SS homologues. Although there was no evident trend for repression, in some treatments the expression of *iglA* and *dotU* was significantly lower at acidic conditions. Future experiments evaluating more time points ideally with addition of proteomic analysis of the putative proteins could help clarify if the lower expression detected is associated with repression or just time of collection.

Avoidance of, or resistance to the respiratory burst is typically required for a successful intracellular life-style, particularly in pathogens that reside in vacuoles and don’t escape into the cytoplasm. Thus, it appears that in order to establish an intracellular niche, *Fno* has to overcome the effect of stress conditions encountered in the hostile environment of the phagosome as it maturates to a phagolysosome. Oxidative stress in the cell arises when the concentration of pro-oxidants like H_2_O_2_ and superoxide anion (O_2_^−^) increase to levels that exceed the cells defense capacity. The production of reactive oxygen (ROS) leads to the damage of intracellular macromolecules such DNA, RNA, protein, and lipids in the cell resulting in bacterial death or bacteriostasis, however intracellular bacteria including *Francisella* have developed oxidative stress defense systems that are designed to detoxify ROS [41, 42] that are generated following respiratory burst.

*Francisella tularensis* LVS is known to survive exposure to 5 mM H_2_O_2_ by induction of several chaperone proteins [43] and IglC [44]. Our data suggest that during oxidative stress, *Fno* T6SS-homologue genes are highly induced, as significantly greater expression of most of the putative *Fno* T6SS genes was detected when exposed to H_2_O_2_ (Fig. 4). Overall our results suggest that the *Fno* T6SS-homologues may play an important role particularly during oxidative stress.

In vitro studies such as those presented in the current study are particularly useful to begin understanding the pathogenesis of important emergent diseases in which methods to study host-pathogen interaction to the molecular or even protein level is hampered by lack of validated tools. However, further research is warranted to clarify the role of pH, reactive oxygen species, and proteases and other enzymes encountered in early and late endosome and phagolysosome; particularly since in the experiments presented in the current manuscript we used them individually, and in-vivo the bacterium may face these challenges simultaneously.

## Conclusions

The results from this study indicate that the expression of several T6SS putative genes in *Fno* changes when the bacterium is exposed to low temperatures and oxidative stress, which suggest a role in pathogenicity; however further clarification of the mechanism used by *Fno* to survive intracellularly is warranted to develop effective therapeutic and prophylactic protocols.

## Methods

### Bacterial strains and growth conditions

*Francisella noatunensis* subsp. *orientalis* (LADL 07-285A) was isolated from naturally infected Nile Tilapia (*Oreochromis niloticus*) in Costa Rica [10]. Bacteria were grown on modified Thayer-Martin agar (Bencton Dickenson BD BBL, Sparks, MD, USA) for 96 h at 25 °C. Broth media (MMH) consisted of Mueller Hinton II cation adjusted, supplemented with 2% IsoVitaleX (BD BBL, Sparks, MD, USA) and 0.1% glucose. Cultures in broth were grown for 24 h in a shaking incubator at 150 rpm and 25 °C.

### Growth curves

Three to five colonies of *Fno* were harvested after incubation on agar media and suspended in 1X phosphate buffered saline (PBS) to achieve a turbidity equivalent to that of a 0.5 McFarland standard. This suspension was diluted 1000-fold (~ 10^5^ CFU/mL) in MMH_._ One hundred μL of inoculated MMH were added to 12 different wells of a Nunc Edge untreated clear flat-bottom 96-well plate with standard microplate lid (ThermoFisher). The Cytation 5 (Biotek) 96-well plate reader was used to obtain optical density measurements at 600 nm, every hour for 96 h. Plates were incubated in the plate reader at 25 °C or 30 °C using a double orbital continuous 3 mm shaking amplitude. Wells on the edges of the plate were not used as experimental wells and were instead filled with MMH as negative control.

In some experiments, *Fno* was collected from exponential (48 h) or stationary (72 h) growth phases for gene expression analysis. Bacteria were suspended in RNAProtect Bacteria Reagent (Qiagen) and nucleic acid was extracted from 500 μL (exponential) or 250 μL (stationary) cultures.

### Bacterial survival to oxidative or acidic stress

*Francisella noatunensis* subsp. *orientalis* culturability under oxidative or acidic stress was investigated following published protocols with modifications [44–46]. Briefly, bacteria was grown overnight in 10 mL of MMH at 25 °C, and used to inoculate 200 mL of MMH. Bacteria were then incubated for another 24 h at the same conditions until exponential phase. Bacteria were washed (centrifugation at 3500 g/10 min and re-suspension in 1XPBS) three times and the pellet suspended in MMH at 0 mM, 0.1 mM, 1 mM, and 5 mM of H_2_O_2_, or MMH at pH 4.0, 6.4, and 7.0. The cultures were incubated statically for 1, 6, 12, and 24 h at 25 °C. At each time point aliquots were removed, and ten-fold dilutions were made in PBS. Different dilutions were then inoculated on MTM II agar plates for CFU quantification. At 1, 6 and 24 h time points, bacterial aliquots were pelleted and suspended in RNAProtect Bacteria Reagent (Qiagen) for gene expression studies.

### Primer design and PCR efficiency

Reverse transcription quantitative PCR was done following protocols by [47]. The sequences of all primers used in this study are listed in Table 2. The *Fno* LADL sequenced pathogenicity island (NC_023029.1) was used to designed primers of putative virulence genes with homology to the *F. tularensis* T6SS, including *vgrG*, *iglA*, *iglB*, *pdpB*, and *dotU* genes. Primer efficiencies were determined using 10-fold dilution series of cDNA and genomic DNA as template for qPCR reactions [44].

**Table 2:**
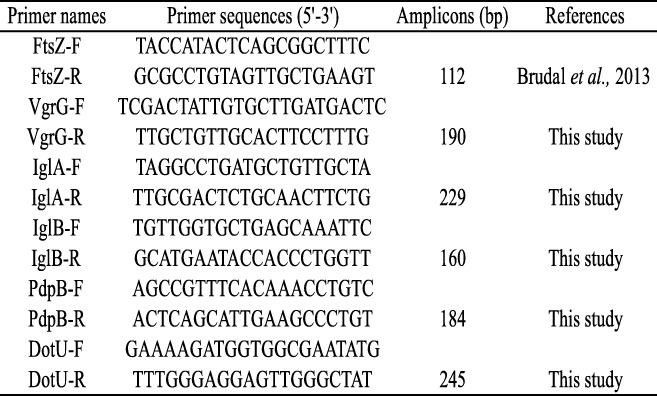
Primers used in this study

### RNA extraction and gene expression

Total RNA was isolated using RNeasy Mini Kit (Qiagen) according to the manufacturer’s instructions. A 15-min on-column DNase digestion with RNase-Free DNase (Qiagen) was performed to ensure removal of contaminating genomic DNA as suggested by the manufacturer. RNA concentration and purity, determined by 260/280, were measured with a spectrophotometer (Cytation 5, Biotek). Reverse transcription of one ug extracted RNA in 20 μL reactions was performed using the Superscript III First Strand Synthesis System (Thermofisher) and oligo dT primers according to the manufacturer’s instructions. A control with omitted reverse transcriptase was performed for each extraction to check for the presence of contaminating genomic DNA. After reverse transcription, the samples were used as templates for reverse transcription quantitative PCR.

### Reverse transcription quantitative PCR (qRT-PCR)

In order to quantify and compare the expression levels of the T6SS-homologue genes, relative quantification by qRT-PCR was made using a reference gene (housekeeping gene) *ftsZ* for the normalization [44] in a QuantStudio 5 Real-Time PCR System (Thermofisher). The PCR mixture contained 1 μL of template cDNA, 1X SYBR Green PCR Master Mix (Applied Biosystems) and 20 μM of the appropriate forward and reverse primers (Invitrogen). The thermal cycling conditions for the PCR were as follows: 1 cycle at 95 °C 10 min, 45 cycles of amplification at 95 °C for 15 s and annealing at 60 °C for 1 min [44]. The data were collected during each elongation step. Melting curve analysis consisting of 1 cycle at 95 °C for 30 s, 55 °C for 30 s, and 95 °C for 30 s was also performed after SYBR Green PCR to check the specificity of the amplification products [44]. Negative (DEPC-treated H_2_O) and no-reverse transcriptase controls were included in each run. All qPCR were assayed on every biological replicate (*n* = 3) and each sample was run in triplicate.

Relative gene expression of *vgrG*, *iglA*, *iglB*, *pdpB*, and *dotU* was calculated using the values obtained from *ftsZ* as a normalization factor. For the expression during growth kinetics the exponential or stationary growth phase at 25 °C/30 °C was used as a calibrator for all genes using the 2^-∆∆Ct^ method [48]. For expression during oxidative and acidic stress, the 0 h of incubation at pH 7.0 and 0 mM H_2_O_2_ were used as calibrator for all genes.

### Statistical analysis

The SAS® (Version 9.4, SAS Institute, Cary NC) GLM procedure was used to analyze the data. All comparisons where considered significant at *p* ≤ 0.05.

#### Growth curve comparison at different temperatures

The SAS® Version 9.4 Proc Mixed was used to analyze the data as a repeated measures analysis of variance. Factors in the model included Temperature (25 °C, 30 °C), Time (1 – 90 h), and the Temperature by Time interaction. The random effect in the model was Replicate (*n* = 12) within Temperature. The response variable was optical density (OD). When terms were significant, post hoc analyses were conducted with pairwise T-test comparisons of least-squares means.

#### Gene expression at different temperatures and growth curve

A 2 X 2 factorial arrangement of treatments was used. Factors in the model Growth (Exponential, Stationary) and Temperature (25 °C, 30 °C). The Response variables were ∆Ct and ∆∆Ct. To stabilize variance terms when necessary, data were adjusted by adding constants to the entire distribution in order to log-transform the data. When overall significance was detected for main effects or interaction effects, post hoc comparisons were conducted with pairwise t tests of least-squares means.

#### Culturability at different pH’s

A 7 X 3 factorial arrangement of treatments was used. Factors in the model included Time (0, 1, 6, 12, 24, 48, 96) and pH (4, 6.4, 7). The response variable was percent survival. To stabilize variance terms when necessary, data were adjusted by an arc sine (inverse sine) transformation. When overall significance was detected for main effects or interaction effects, post hoc comparisons were conducted with pairwise t tests of least-squares means.

#### Gene expression at different pH’s

A 5 X 3 factorial arrangement of treatments was used. Factors in the model included Time (0, 1, 6, 12, 24) and pH (4, 6.4, 7). The Response variables were ∆Ct and ∆∆Ct. To stabilize variance terms when necessary, data were adjusted by adding constants to the entire distribution in order to log-transform the data. When overall significance was detected for main effects or interaction effects, post hoc comparisons were conducted with pairwise t tests of least-squares means.

#### Culturability at different concentrations of hydrogen peroxide

A 5 X 4 factorial arrangement of treatments was used. Factors in the model included Time (0, 1, 6, 12, 24) and H_2_O_2_ concentration (0, 0.1, 1, 5). The response variable was percent survival. To stabilize variance terms when necessary, data were adjusted by an arc sine (inverse sine) transformation. When overall significance was detected for main effects or interaction effects, post hoc comparisons were conducted with pairwise t tests of least-squares means.

#### Gene expression at different concentrations of H_2_O_2_

A 5 X 4 factorial arrangement of treatments was used. Factors in the model included Time (0, 1, 6, 12, 24) and H_2_O_2_ concentration (0, 0.1, 1, 5). The Response variables were ∆Ct and ∆∆Ct. To stabilize variance terms when necessary, data were adjusted by adding constants to the entire distribution in order to log-transform the data. When overall significance was detected for main effects or interaction effects, post hoc comparisons were conducted with pairwise t tests of least-squares means.

## Additional file

Additional file 1: **Figure S1.** Growth curves for *Francisella noatunensis* in broth incubated at 25 °C and 30 °C for 96 h. The error bars represent the standard deviation of twelve replicate wells from three independent experiments. (PDF 55 kb)

## Abbreviations

*Fnn*: *Francisella noatunensis* subsp. *noatunensis*
*Fno*: *Francisella noatunensis* subsp. *orientalis*
H2O2: Hydrogen peroxide
MMH: Modified mueller-hinton
PBS: Phosphate buffered saline
PI: Pathogenicity island
qRT-PCR: Reverse transcription quantitative PCR
T6SS: Type VI secretion system
