# Supplementary figures and images for: Gene expression of putative type VI secretion system (T6SS) genes in the emergent fish pathogen Francisella noatunensis subsp. orientalis in different physiochemical conditions
Source: BMC Microbiol. 2019 Jan 21;19:21. doi: 10.1186/s12866-019-1389-7 (PMC6341738; doi:10.1186/s12866-019-1389-7)

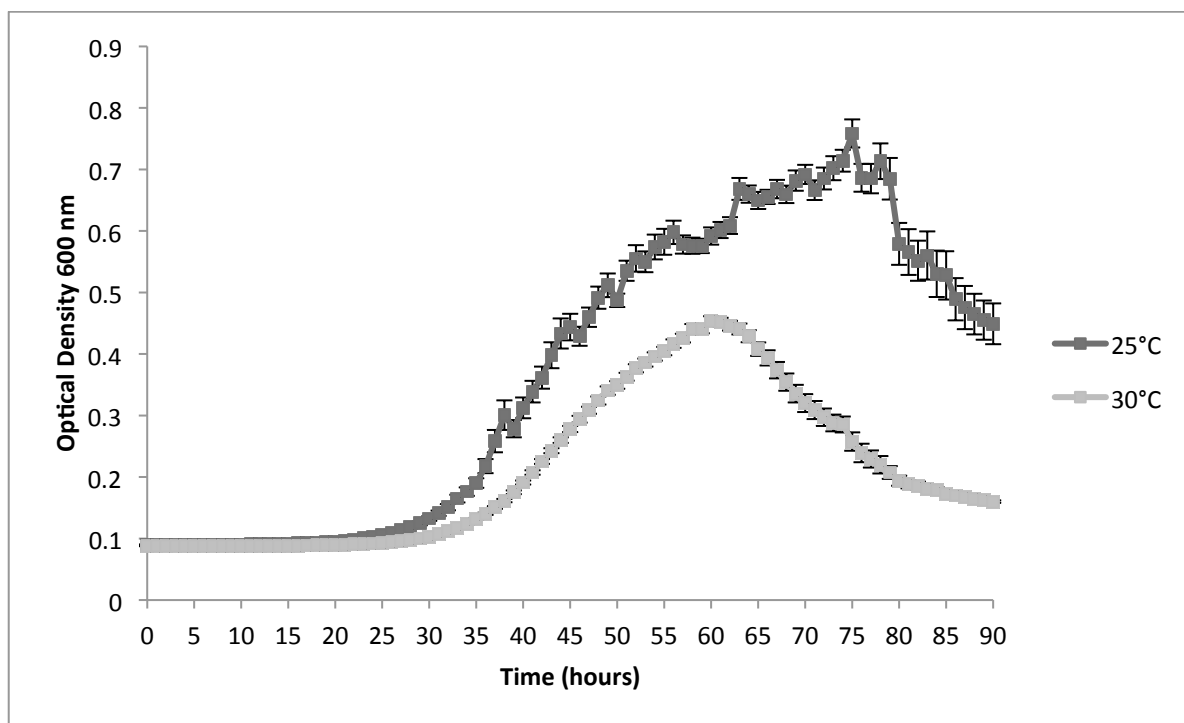

Supplement: Supplementary file 1 — Figure S1. Growth curves for Francisella noatunensis in broth incubated at 25 °C and 30 °C for 96 h. The error bars represent the standard deviation of twelve replicate wells from three independent experiments. (PDF 55 kb) [file 12866_2019_1389_MOESM1_ESM.pdf]
